# Effects of Marine Toxins on the Reproduction and Early Stages Development of Aquatic Organisms

**DOI:** 10.3390/md8010059

**Published:** 2010-01-19

**Authors:** Vítor Vasconcelos, Joana Azevedo, Marisa Silva, Vítor Ramos

**Affiliations:** 1 Faculty of Sciences, Porto University, Rua do Campo Alegre, 4169-007 Porto, Portugal; 2 Marine and Environmental Research Center, CIIMAR, CIMAR-LA, Rua dos Bragas, 289, 4050-123 Porto, Portugal; E-Mails: joana_passo@hotmail.com (J.A.); marisasilva17@gmail.com (M.S.); bioramolas@hotmail.com (V.R.)

**Keywords:** marine toxins, reproduction, larval development, early stages

## Abstract

Marine organisms, and specially phytoplankton species, are able to produce a diverse array of toxic compounds that are not yet fully understood in terms of their main targets and biological function. Toxins such as saxitoxins, tetrodotoxin, palytoxin, nodularin, okadaic acid, domoic acid, may be produced in large amounts by dinoflagellates, cyanobacteria, bacteria and diatoms and accumulate in vectors that transfer the toxin along food chains. These may affect top predator organisms, including human populations, leading in some cases to death. Nevertheless, these toxins may also affect the reproduction of aquatic organisms that may be in contact with the toxins, either by decreasing the amount or quality of gametes or by affecting embryonic development. Adults of some species may be insensitive to toxins but early stages are more prone to intoxication because they lack effective enzymatic systems to detoxify the toxins and are more exposed to the toxins due to a higher metabolic growth rate. In this paper we review the current knowledge on the effects of some of the most common marine toxins on the reproduction and development of early stages of some organisms.

## 1. Main Marine Toxins Produced by Bacteria and Phytoplankton

Many species of marine microorganisms produce toxins that have significant impact in other species such as invertebrates, vertebrates and in many cases, human populations. Among other groups, Bacteria, Cyanobacteria, Diatoms, Crysophytes and Dinoflagellates may synthesize compounds that are kept in most cases within the cells, but affect other organisms when the toxins are ingested or when toxic blooms collapse and expose the toxins to organisms that live in the same environment. Toxins may also be found in higher trophic levels due to bioaccumulation along food chains. The main toxins produced by bacteria and phytoplankton are summarized in Table 1. Most of the toxins have neurotoxic properties, except for the hepatotoxin nodularin that is produced by a species of cyanobacteria commonly found in brackish waters.

**Figure 1:**
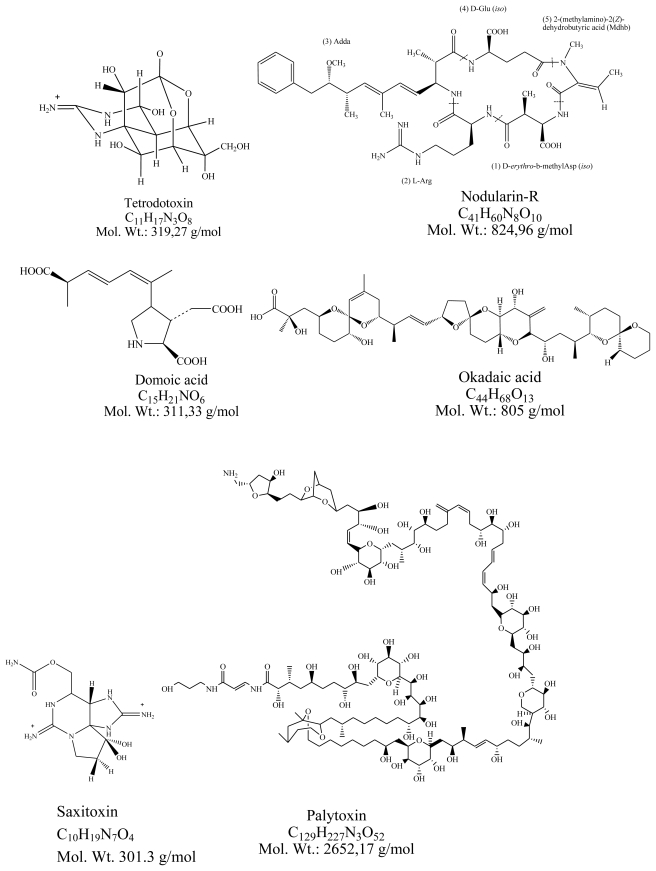

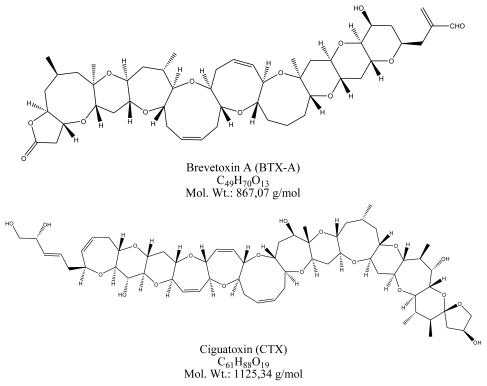
Structures of tetrodotoxin, nodularin, domoic acid, okadaic acid, saxitoxin palytoxin, brevetoxin and ciguatoxin.

Tetrodotoxin (TTX) (Figure 1) is one of the most potent natural occurring low molecular weight marine neurotoxin, being responsible for human food poisonings, including death. TTX receives its name after the puffer fish family Tetraodontidae. This neurotoxin blocks the voltage-gated sodium channels on the surface of nerve membranes. Chemically it is a tricyclic structure, non-proteic basic alkaloid that consists of a positively charged guanidinium group. TTX mimics the hydrated sodium cation, it blocks the TTX-sensitive sodium channels inhibiting the generation and propagation of action potential by voltage-gated sodium channels in electrically excitable cells. These properties are of very high value to the pharmacological and physiology research of identification, isolation, purification, and characterization of voltage-gated sodium channels [1,2]. Tetrodotoxin is found in fugu fish and causes human lethal intoxications by consumption of the fish when not correctly prepared and is found in many organisms, being bacteria the producing organisms in the aquatic environment. Tetrodotoxin is also produced by many bacteria species isolated from the intestine of the Puffer fish (*Fugu vermicularis radiatus*) such as *Vibrio* [3], *Serratia marcescens* [4,5] and *Microbacterium arabinogalactanolyticum* [4] (Table 1). Moreover, the toxin was reported in invertebrate species such as starfish [6] and crabs [7] and in several terrestrial vertebrates such as newts and tree frogs from North America [8].

Nodularin (NOD) (Figure 1) is a cyclic pentapeptide produced by the cyanobacterium *Nodularia spumigena* that inhibits protein phosphatases and causes liver disruption that may result in a severe liver failure and death by hipovolemic chock [9]. Protein phosphatases (PP) are enzymes responsible for the dephosphorylation of various proteins in a cell. This is extremely important since protein phosphorylation and dephosphorylation is required for the regulation of a large number of cellular activities and required in several processes during embryonic development. Both PP2A and PP1 were potently inhibited (IC_50_ = 0.026 and 1.8 nM, respectively) by nodularin, whereas PP2B was inhibited to a lesser extent (IC_50_ = 8.7 μM). Nodularin had no apparent effect on PP2C.

Domoic acid (DA) (Figure 1), an amino acid produced by diatoms of the genus *Pseudo-nitzschia* is a glutamate receptor agonist. DA is a rigid analog of the excitatory amino acid glutamate [10]. Studies on the possible role of bacteria associated to *Pseudo-nitzschia* on the production of DA revealed that bacteria are not responsible but eventually can provide precursors that enhance DA production [11]. No data on the chronic effects of DA in animals are available Ingestion of DA contaminated shellfish by humans produces the Amnesic Shellfish Poisoning (ASP).

Okadaic acid (OA) (Figure 1) is a polyether produced by *Dynophysis* species, has a neurotoxic effect and inhibits PP1 and PP2A [12]. Ingestion of OA contaminated shellfish by humans produces the Diarrheic Shellfish Poisoning (DSP).

Saxitoxins (STX) represent a group of neurotoxic alkaloids produced both by dinoflagellates and freshwater cyanobacteria (Figure 1). They include pure saxitoxin (STX), neosaxitoxin (neoSTX), the gonyautoxins (GTX) and decarbamoylsaxitoxin (dcSTX). STX act on the voltage-gated sodium channels of nerve cells, preventing normal cellular function and leading to paralysis [13]. Ingestion of shellfish contaminated with STX produces the Paralytic Shellfish Poisoning (PSP) in humans.

Palytoxin is (PTX) is a large, very complex molecule (Figure 1) with a long polyhydroxylated and partially unsaturated aliphatic backbone, containing 64 chiral centers [14]. This latter feature, coupled with the presence of eight double bonds able to exhibit cis/trans-isomerism means that PTX can have more than 10^21^ stereoisomers [15]. PTX seems to bind to the Na^+^/K^+^-ATPase extracellularly and therefore inhibit the active transport of Na^+^ and K^+^ across the cell membrane by transforming the pump into a non-specific permanently open ion channel. The membrane depolarization generated and the massive increase of Ca^2+^ in the cytosol [16] interferes with some vital functions of cells. An altered concentration of intracellular cations, in particular a calcium increase, is generally associated with cell death [17].

Brevetoxins (BTX) (Figure 1) are a group of cyclic polyether compounds produced by the dinoflagellate *Karenia brevis*. BTX are neurotoxins that bind to voltage-gated sodium channels in nerve cells, leading to disruption of normal neurological processes and causing the illness clinically described as Neurotoxic Shellfish Poisoning (NSP). There are several variants, being PbTX-3 one of them, a congener of type B brevetoxins (one of 10) [18].

Ciguatoxin (CTX) (Figure 1) is a neurotoxin produced by the dinoflagellate *Gambierdiscus toxicus* that accumulates in the skin, head, viscera and roe of many species of reef fish. CTX are potent, lipophilic sodium channel activator toxins which bind to the voltage sensitive sodium channel on the cell membranes of all excitable tissues [19].

Most of the toxins produced by marine microorganisms such as tetrodotoxin, saxitoxins, palytoxin, brevetoxins or ciguatoxin (Figure 1) have Na^+^ or K^+^ channels as targets. Nevertheless, while most of them block the channels preventing the depolarization of the membranes and stopping the nervous influx, palytoxins is a pore forming toxin that opens the flux of Na^+^/K^+^ pumps over stimulating the axons and causing death by muscle fatigue.

Chemically, apart from the pentapeptide nodularin, the other marine toxins are polyethers or alkaloids (Table 1). The toxins produced by marine microorganisms usually affect other organisms either if the producing organisms occur as a bloom or if they are accumulated by vectors and transferred along food chains. This seems to be the case of TTX (Figure 1). The other toxins produced by primary producers are known also because they cause severe intoxications in humans, sometimes with lethal effects.

Although many of these toxins are of tropical origin, they have been found in more temperate areas (Atlantic, Mediterranean), suggesting an invasive behavior, due to climatic reasons or more efficient routes of dissemination.

## 2. Effects of Marine Toxins in Aquatic Invertebrate Larval Development

The classification of the marine toxins based on the effects on mammals in hepatotoxins and neurotoxins is not adequate for the effects that they produce in invertebrate and vertebrate larval development and reproduction. This may be due not only to the different physiology of these groups of organisms but also because the toxins are many times characterized only by a single physiological/molecular activity and they may act in different targets.

### 2.1. Tetrodotoxin

Although information and research on TTX in the chemistry and biological field is abundant, there is a lack of experiments testing its lethal and sublethal effects in the early development of larvae. The larvae of *Parasarcophaga argyrostoma* (Diptera, Sarcophagidae) can be used as a toxicological biotest to detect marine toxins with the purpose of monitoring big-sized fish [20]. In such a study, ten larvae were left overnight on a 5 g test sample of moray eel (*Gymnothorax javanicus*) flesh with a determinate known toxicity. A decrease in the mobility, complete immobilization and eventually death was observed, which enabled the classification of TTX as toxic. However, surprisingly TTX seemed to be less poisonous to larvae than for man [20]. McMachon *et al*. [21] studied the effect of TTX on the larval development of penaeid shrimp (*Metapenaeus ensis*). TTX injections inhibited the heartbeat of later juvenile instars, while no inhibitory effect was observed in the heartbeat frequency of larval and early juvenile instars. These findings led the authors to suggest that neurogenic regulation *via* cardiac ganglia arise later in the development of the crustacean [21].

### 2.2. Nodularin

NOD produced by *Nodularia* can vary accordingly to environmental conditions and the dominance of NOD-producing *N. spumigena* strains [22]. Although the concentration of NOD varies, values of 14–17 μg NOD L^−1^ are common in the Baltic Sea [23]. Zooplankton, being a group of organisms that feed directly of phytoplankton, may be considered as the preferential target of NOD. In fact it seems that NOD affects either in the reproduction success or the embryo development of several zooplankton species, depending of the way the organisms are exposed to the toxin. Koski *et al*. [24] found that *Eurytemora affinis* mortality was high when the copepods were fed with NOD-producing *Nodularia* (Table 2). Mortality of copepods fed only a NOD producing strain was 44–72% compared to 6–29% when fed a non-NOD-producing *Nodularia*. Egg production was not affected in copepods by food concentration if copepods were fed NOD or non-NOD strains for 48 h.

With NOD *Nodularia*, hatching success was significantly lower than with other food species. None of the eggs produced with a NOD diet hatched, but in mixtures success was comparable to other diets. The copepod was not able to reproduce on a diet of *Nodularia*, irrespective of the presence of NOD. The nutritional quality of *Nodularia* was not high enough to support viable egg production.

Later Koski *et al*. [25] studied the reproductive behavior of two copepod species common in the Baltic Sea, *Acartia bifilosa* and *Eurytemora affinis*, fed an assemblage dominated by cyanobacteria producing NOD. Irrespective of the NOD concentration in the food (9.2–12 μg NOD L^−1^), egg production in *A. bifilosa* was observed, showing that copepods in the Baltic may feed, survive and reproduce in plankton communities dominated by toxic cyanobacteria. Ojavier *et al*. [26] studied the effects of a *Nodularia* strain producing NOD on the copepod *Eurytemora*, showing that males and females were affected by the toxin, with males being more sensitive (TL_50_ = 2.5 days) than females that showed a mortality rate lower than 30% at the end of day 6 of exposure. Kozlowsky-Suzuki *et al*. [27] studied the effects of several toxic algae and cyanobacteria on the survivorship and reproduction of male and females of the copepod species *Acartia tonsa. A. tonsa* did not fed on *N. spumigena* on a 24 h experiment. A 5 day experiment showed that a diet on *N. spumigena* producing NOD completely inhibited egg production by *A. tonsa*. This was confirmed by histology analysis of the gonads of *A. tonsa* that were decreased in size in NOD treatments compared to control copepods. Degradation of cytoplasm and cell fragmentation in OS3 and lack of OS4 confirmed the gonad atresia in females at the end of day 6. Survivorship was not significantly affected in spite of the severe food limitation.

These results, although seemingly contradictory, show that although NOD affects the reproduction and development of zooplankton, it depends on the route of exposure, if the organisms are exposed to pure *N. spumigena* culture or mixtures and on the sex of the organisms (in case of copepods). The hatching success of the eggs is more dependent on the food condition during production than on the quality of the media surrounding the eggs. A negative effect on egg hatching is mediated *via* accumulation of toxins in the female and inhibition or disturbance of the embryogenesis. *E. affinis* is able to survive but not reproduce in a mono-specific bloom of *Nodularia* if the strain is NOD negative. If NOD is present the mortality rate is high.

Other invertebrates such as the amphipod *Gammarus zaddachi* were studied. Korpinen *et al*. [23] studied the effects of NOD and NOD-producing *Nodularia* on the survival and reproduction of adult *G. zaddachi.* They were exposed to different concentrations of extracts of *Nodularia* and the highest concentration with 101 μg NOD L^−1^ produced a LT_50_ of 1 day, while 41 μg NOD L^−1^ produced a LT_50_ of 2.5 days and the lowest concentration of 30 μg NOD L^−1^ did not cause any mortality. Exposure to pure NOD at concentrations of 12 and 50 μg NOD L^−1^ did not cause any mortality. Exposure to dissolved NOD did not result in a significantly higher number of egg abortions compared to the control, while extract of *N. spumigena* with a NOD concentration of 30 μg NOD L^−1^ caused a number of abortions significantly higher than control or comparable to pure NOD treatment. Extracts seem to be more toxic than pure toxin at equivalent NOD concentrations. Purified NOD and *N. spumigena* extracts with NOD did not have a significant effect on egg hatching rates. Eggs and juveniles of *G. zaddachi* accumulated more NOD than adults with juveniles accumulating NOD (~2 ng NOD μg^−1^ C), two orders of magnitude more than eggs. NOD seems to be taken up by oral consumption more than by absorption. So it seems that at concentrations normally found in the Baltic, there seems to be no acute effect of NOD to the gamaridae.

Invertebrates seem to have a defense mechanism against NOD [28]. Four week old *Artemia* exposed to pure NOD (0.5 μg NOD L^−1^) showed that after 24 h both soluble glutathione-s-transferase (sGST) and microsomal-s-transferase (mGST) were elevated significantly relative to the control, indicating an active detoxication mechanism [28].

### 2.3. Domoic acid

Diatoms, such as *Pseudo-nitzschia* species are natural food used by zooplankton in marine systems. The potential production of DA by *Pseudo-nitzschia* species may represent a hazard to these organisms. Nevertheless, Maneiro *et al*. [29] studied the effect on survivorship of *Acartia clausii* that was fed with a mixture of *Pseudo-nitzschia* sp and *Tetraselmis*, showing that the inclusion of the DA producer in the diet did not significantly increase the copepod mortality. The toxins ingested did not affect mortality, feeding behavior, egg production and egg hatching at ecologically significant DA concentrations (Table 2). Also, Liu *et al*. [30] studied the effects of a formulated feed spiked with DA on *P. maximus*. The gonad index was not significantly different from the control. No significant changes were found as well in spermary, ovary or fertilized eggs after DA exposure. DA excretion seems to be more efficient that DA accumulation. The effects of a 25 day exposure to DA (30 and 50 ng DA mL^−1^) on fertilized eggs of *Pecten maximus* revealed that eye spotted larva accumulated a maximum of 5.21 ± 0.49 pg ind^−1^ (3.26 ± 0.31 μg DA g^−1^) when exposed to 50 ng DA mL^−1^ [28]. The final shell length in exposed organisms to DA was significantly smaller than control. Larval survivorship after the 25 day was also affected and different from control, but no significant differences were found between the 30 and 50 ng concentrations. No significant effects of DA on larval activity and no acute pathological effects were found. Effects of DA in juvenile king scallops (*P. maximus*) was studied and a maximum of 302.5 ng DA g^−1^ was accumulated during 18 days [31]. No significant effects of exposure to DA were observed on feeding rate on microalgae, shell valve activity, righting response or secretion of bissus threads. However, negative impacts on growth rate and survival were found on scallops exposed to DA spiked feed. After 38 and 68 days of DA feed, larva had higher death rate than control animals. Most of the studies show that exposure to DA causes little or no effects of survival and reproduction of several invertebrate species (mollusks and crustaceans). This absence of effects may be due to fact that the molecular target of DA is the glutamic receptors in the brain cells that may be absent or physiologically different in invertebrates and vertebrates.

### 2.4. Okadaic acid

OA is produced by several dinoflagellate species that may be used as food by zooplankton and larvae of invertebrate. Size of the cells may be more deterrent of ingestion than toxin content. Perez and Suikin [32] showed that the larvae of five crab species ingested the small *Prorocentrum minimum* producer of OA, but did not ingest *P.* hoffmannianum, also a producer of OA. This was explained by the larger size of the second dinoflagellate species and also by the fact that they form large chains with mucus. Assays on the palatability and toxicity of *Prorocentrum lima* producer of OA and DTX in *Artemia* showed that cell free medium did not affect hatching rate of *A. salina* [33]. A similar result was found when analyzing the hatching rate of eggs of the copepod *Euterpina acutiformis* exposed to cell free extracts of *A. minutum* [34]. The protective cyst wall might have prevented the toxin from penetrating the cysts. Nevertheless, 50% of cell free medium caused a high lethality to *Artemia* metanauplii within 24 h. Ingestion of cells also caused high mortality of nauplii, especially in high density treatments (Table 2). The swimming behavior of nauplii that ingested *P. lima* was affected, they lost balance, failed to swim and sank to the bottom before dying. It seems that early stages of the invertebrate life cycle are able to handle OA producing strains, but swimming stages are prone to suffer lethal effects.

### 2.5. Saxitoxins

The effects of STX on invertebrate reproduction and development have been studied mostly on crustacean (*Artemia* and several crab and lobster species) larvae. Effects range from lethal in *Artemia*, to sublethal in crab and lobster larvae. The impact of STX may be seen either in direct physiological terms (changes in oxygen consumption, changes in reproduction success) or by changes in behavior (altered vertical migration patterns).

Effects of exposure of *A. salina* nauplii (1 day), metanauplii (4 days) and adults (10 days) to cultures of *Alexandrium catenella, A. minutum* and *A. tamarense,* producers of STX showed that survival of metanauplii decreased more rapidly that of other development stages [35]. The toxic *Alexandrium* spp. at a density of 2,000 cells mL^−1^ was lethal to *Artemia* and can restrain the feeding of the crustaceans (Table 2). Metanauplii was the most sensitive stage. These results showed that STX producing strains of dinoflagellates can induce lethality to populations of *Artemia* at ecologically relevant densities.

In terms of physiological changes that may interfere with invertebrate reproduction, Robineau *et al*. [36] studied the effects of *Alexandrium excavatum*, producer of STX (10.5 × 10^−5^ μg STX eq. cell^−1^) on larvae of American Lobster (*Homarus americanus*). Lobster larvae were tested at stages I to IV. Larvae were exposed to toxic dinoflagellates *via* intoxicated zooplankton. Survivorship was high in most larval stages: 100% in stage I, 85% in stage II, 57% in stage III and 94% in stage IV. The absence of changes in behavior and low mortality rates indicate that lobster larvae seem to be immune to STX.

*Cancer magister*, *C. oregonensis*, *Hemigrapsus oregonensis* and *Rhinolithodes wosnessenskii* larvae were fed with five species of dinoflagellates (producers of STX and non producers) alone and in mixed algal diets [37]. The control non STX *Prorocentrum micans* was ingested by all larvae at all observed times. On the other side, the STX producer *A. fundyense* was only ingested by *C. oregonensis* but a rapid decline was shown with 0% ingestion at 48 h. Both SXT and no STX producer strains of *A. tamarense* were almost never ingested by any species at any time. Larvae fed *A. fundyense* and no STX *A. tamarense* died at the same time as unfed controls showing that both strains did not contribute nutritionally nor had a toxic effect for all *Cancer* species. If *Alexandrium* was mixed with *P. micans* all cells were ingested by all crab species [37]. For larvae to discriminate among prey they must respond to prey-associated cues, either chemical or mechanical stimuli. The failure of larvae to ingest both STX and non STX producing *Alexandrium* strains does not support the idea that toxins prevented the ingestion by larvae. The results do not support the role of a water-borne chemical stimulus either positive or negative. The non ingestion of toxic cells of *Alexandrium* prevented any toxic effect. Perez and Suikin [32] studied the use of several dinoflagellate producing STX and OA on the survivorship of six species of crab larvae. Dinoflagellate cell density was 10^3^ cells mL^−1^. Zoea larvae of *Cancer magister, C. oregonensis, C. productus, C. gracilis, Hemigrapsus oregonensis* and *H. nudus*, readily ingested a toxic strain of *Alexandrium andersonii* producing STX while did not ingest the non toxin producer *A. tamarense*. Concerning lethal effects, larvae of *H. nudus* and *H. oregonensis* fed the toxic *A. andersonii* had a significantly lower survivorship, compared to unfed crabs. The results do not support previous suggestion that *Alexandrium* spp. are generally unpalatable to larval crabs. There is no consistent pattern of prey discrimination based on taxonomic affinity, toxicity or predator/prey size relationships. Thus, it is clear that toxicity is not the only basis of discrimination.

Sublethal effects produced by STX were studied using larvae of *C. oregonensis* exposed to cells of *A. tamarense* (no STX) and *A. fundyense* (STX) and the latter ones caused a decrease on oxygen consumption rates 6 hours after the beginning of the experiment [38]. On the contrary, larvae of *C. magister* exposed to both strains showed a significant reduction of oxygen consumption compared to sea water control. Both crab species if exposed to filtrate of *A. fundyense* from cultures with more than 5 × 10^2^ cell mL^−1^ showed a significant reduction in oxygen consumption. So it seems that the effects of STX depend on the target species, being some invertebrate more resistant than others to the toxins.

Some effects of the exposure to the toxins may be expressed as changes in behavior. This might have a significant impact on the biology of the affected population, exposing them to predators or difficulting the search for food. When larvae of *C. oregonensis* were exposed to STX *A. fundyense* cells, they showed much reduced upward movement [38]. The majority remained in the middle of the chamber indicating a negative geotaxis. Control organisms and those exposed to no STX *A. tamarense* showed upward movement, typical of larvae exhibiting negative geotaxis. *C. magister* had a similar negative geotaxis response for both strains. Reduced rates of oxygen consumption in larvae exposed to toxic *A. fundyense* could be due to reduction in base metabolic rate or to reduced locomotion [38]. The decline of locomotion of *C. oregonensis* larvae exposed to *A. fundyense* may be due to initial ingestion of toxic cells. Vertical movements result from the interaction between responses to orientation stimuli and levels of locomotion that are regulated by intrinsic and extrinsic cues. In negative buoyant crab larvae reduced locomotion will produce downward movement. Thus when larvae encounter a bloom of toxic algae, respond by reducing locomotion, they will sink to greater depths moving out of the bloom. The consequences between alternating favorable and unfavorable feeding environments on larval survival and growth are not known. Periodic exposure to toxins may have obvious negative consequences.

Larvae of many species of crabs change their behavior in response to directional cues by varying their locomotory activity which in turn regulates their depth and influences vertical migration in the pelagic environment. Patterns of vertical migration have two major consequences for larval survivor and ultimate recruitment to the adult habitat. First, the vertical distribution of larvae at various stages of development can profoundly influence dispersal. Secondly, a pattern of behavior that induces upward movement in the water column soon after hatching. It also promotes vertical migrations at various time scales that may increase the probability of these plankton larvae will encounter dense patches of meso-zooplankton prey that they may require to feed effectively.

### 2.6. Ciguatoxin

Not much is known about the toxic effects of CTX to invertebrate development and reproduction, possibly because the final targets of the toxins are reef fish. The effects of CTX from *Gambierdiscus toxicus* on adult *Artemia* sp. fed different cell numbers (2 to 1,000 cells) produced a LC_50_ of 2.8 to 104.5 cells depending on the strain of *G. toxicus* (Table 2) [39]. Comparing toxicity results of *Artemia* and mice, the crustacean was 100 times more sensitive using the same strains. These results are interesting because they show a high lethality rate of CTX exposed *Artemia*, showing that other organisms may be better vectors of the toxin. Probably phytophagous reef fish have a higher resistance to CTX, being efficient vectors of this toxin.

The effects of the marine toxins in early life stages of invertebrates do not show a define pattern accordingly to effects in vertebrate or to the chemical groups of the toxins. Most of the studied toxins may cause lethal effects to invertebrate larvae at ecologically relevant concentrations or cell density. TTX and CTX are those that have been less studied from this point of view maybe because the final vector of the toxin is usually a fish and so vertebrate studied are more common. Nevertheless, both toxins show lethal effects to *Artemia* nauplii, showing that toxin uptake and bioaccumulation may have other vectors apart from zooplankton. NOD affects the reproduction and development of zooplankton, depending on the route of exposure, if the organisms are exposed to pure *N. spumigena* culture or mixtures and on the sex of the organisms (in case of copepods). A negative effect on egg hatching is mediated *via* accumulation of toxins in the female and inhibition or disturbance of the embryogenesis. Zooplankton seems to survive, but not reproduce in a mono-specific bloom of *Nodularia* if the strain does not produce NOD. Since usually blooms may contain other non toxic species, impact on the zooplankton survivorship and reproduction may be lower. DA causes little or no effects of survival and reproduction of several invertebrate species (mollusks and crustaceans). This absence of effects may be due to fact that the molecular target of DA is the glutamic receptors in the brain cells that may be absent or physiologically different in invertebrates and vertebrates. It seems that early stages of the invertebrate life cycle are able to handle OA producing strains however swimming stages are prone to suffer lethal effects.

## 3. Effects of Marine Toxins in Aquatic Vertebrate Early Development

Fish may be exposed to marine toxins during the different stages of their reproduction process, as adults during sexual maturation, as eggs and as larvae. In all these phases, the impact of a single toxin is different because the levels of exposure and the way the toxin may find their target is also different. Most of the times the contact with the toxins might be directly, *via* food or during collapse of phytoplankton blooms when the toxins are dissolved in the environment. Comparative studies on the effects of the various marine toxins on egg hatching and embryo development are very difficult due to the fact that several model fish species are used (*Danio rerio* and *Oryzias latipes*) and different routes of administration (microinjection of pure toxin, exposure to pure toxin or to raw extracts) (Table 3).

### 3.1. Domoic acid

In order to study effects of DA in *D. rerio* embryos, fish eggs were microinjected with doses ranging from 0.12 to 17 μg DA g^−1^ egg weight. Exposure to DA reduced success of egg hatching −EC_50_ = 0.4–1.2 μg DA g^−1^. At 4.0 μg DA g^−1^ all embryos hatching at 4 days post fertilization (dpf) showed absence of touch reflexes. On the other side no significant differences were found in the heart rate between DA treated and control eggs. Toxic effects induced a reduction in hatching that may be related to the spinal deformities observed. All embryos and larvae (6 dpf) treated with DA showed some rapid and constant uncontrolled pectoral fin movements, compared to control fish [10]. Bejarano *et al*. [40], analyzed data on the deaths of sea lions in California due to DA. Although DA impacts all age classes, the majority of lethal cases have been adult females (47–82% of total annual cases). Large number of female stranding may reflect differences in the structure of populations as well as differences in aggregation patterns during bloom periods. Large females have an increased time spend on the sea, diving for food, increasing the exposure to DA. Also males feed on different grounds compared to those of females.

### 3.2. Okadaic acid

Casarini *et al*. [44] studied the effects of exposure to OA by the use of FETAX (Frog Embryo Teratogenesis Assay–*Xenopus*). This assay measures mortality, delayed growth and embryo deformation. Exposure of embryos at gastrula stage (8 hours post fertilization, hpf) to different concentrations − 0.1 to 10 nM OA, did not cause high mortality rates, with the highest concentrations −1 and 10 nM causing less than 10% mortality after 48 h. On the other side, the growth rate of the embryos was significantly delayed at the concentrations of 0.1 to 10 nM compared to controls. The number of malformed embryos at the end of the assay was also higher and significantly different in the OA treated embryos compared to the controls. Several disorders in the nervous system were observed in the highest concentrations, including rhombencephalon and distorted spinal cord, reduced in size and irregular arrangement of neural cell bodies. Tail skeleton musculature was damaged, the muscle fibers smaller and fewer in number and irregularly distributed compared to the control sections. Apoptotic cells in the rhombencephalon were significantly higher in the two highest concentrations −1 and 10 nM OA compared to the control. Studies of impact of OA (0.1–1 μg OA mL^−1^) and crude extracts containing OA (0.1–0.2 μg OA mL^−1^) in the embryos of *O. latipes* showed that the lethal effects produced by pure toxin and extracts were different, with an LD_50_ for pure OA of 0.52 μg OA mL^−1^, while for the extract the value was 0.17 μg OA mL^−1^ [43]. Intestine was the main target of OA, resulting also a significant increase in liver and digestive tract indicative of volume change, and fluid accumulation as it is shown in rodents [45].

The expression of several genes important during embryonic development of vertebrates–*siamois, engrailed 2, bmp4* and *myf5*–was differently affected after exposure to OA in *X. laevis* [46]. The gene *siamois* was over-expressed in the 11 and 12 gastrulation stages in embryos exposed to OA. The gene *bmp4*–bone morphogenetic protein 4 - was over-expressed as a response of exposure to OA at stages 11, down-regulated at stages 12–17 and increased again after stage 35 (hatching). On the contrary the gene *myf5*–myogenic factor 5 - was up-regulated in all stages up to 35. Effects of exposure to OA seemed to be more stage dependant with the embryonic development, which is more sensitive than larval stages. The damages in the nervous structures and the development defects may be related to the induced *bmp4* down-regulation during neural induction and pattering stages. All the tested genes showed any type of de-regulated expression patterns that may account for previous data using the FETAX assay [46].

### 3.3. Saxitoxins

The toxicity of STX towards eggs and larval stages of fish depends if the toxin is provided in the dinoflagellate cells or as pure toxin. Some works done exposing larvae of several species of marine fish to ecologically relevant densities of cells of dinoflagellates showed that lethal effects may be severe. Gosselin *et al*. [47] exposed larvae and post-larvae of two fish species–capelin, *Mallotus villosus*, and Atlantic herring *Clupea harengus –* to *Protogonyaulax tamarensis* producer of STX (10.5 × 10^−5^ μg STX eq. cell^−1^). Using cells densities of 1,500 cells mL^−1^, they registered a mortality of 92% and 77% for *M. villosus* and *C. harengus*, respectively. Fish exposed to strains producing STX had symptoms of paralysis with erratic swimming, sank to the bottom and died. A non toxin producing dinoflagellate did not produce any of these symptoms. The survivorship of *M. villosus* was concentration dependant, varying from 0 to 92% depending of the dinoflagellates cell density. For *C. harengus* a similar pattern was observed but less pronounced. A similar work studied the effects of *Alexandrium excavatum*, producer of STX (10.5 × 10^−5^ μg STX eq. cell^−1^) on larvae of Atlantic mackerel (*Scomber scombrus*) [36]. Fertilized eggs of *S. scombrus* were hatched and 6–7 day old larvae (herbivorous stage) exposed to cells of *A. excavatum* in densities ranging from 250–1,500 cells mL^−1^. After 16 h all fish larvae exposed to 1,500 cells mL^−1^ died and those exposed to 875 cells mL^−1^ had a 85% death and 250 cells mL^−1^ caused a mortality of 60%. Poisoned fish larvae exhibited abnormal behavior; swam erratically, sank to the bottom and eventually died. These data suggest that marine fish larvae are quite sensitive to STX producing dinoflagellate and that blooms may cause a severe change in fish stocks.

Studies using pure STX may be important to understand the physiological effects of the toxin because they are not masked by other possible secondary metabolites produced by dinoflagellates in whole cell studies. Oberemm *et al*. [41] studied the effects of exposure to STX on embryo development using eggs of zebra fish (*Danio rerio*) and of the amphibian axolotl (*Ambystoma mexicanum*). Embryos were exposed to concentrations ranging from 10–500 μg STX L^−1^. At the highest concentration, embryos of *D. rerio* showed deformations such as lateral and ventral body curvature and edema, after 6 days of exposure. With this concentration–500 μg STX L^−1^ the mortality reached 40% after 21 days of larval development. Also the timing of hatching was delayed in a concentration dependant manner. The effects in *A. mexicanum* were less pronounced, but the time of hatching was also delayed. No chronic effects were detected when the embryos were reared. Lefebvre *et al*. [50] used also *D. rerio* as a model organism to study the effects of STX on sensorimotor function and morphology of their larvae. Embryos were exposed to concentrations ranging from 229–481 μg STX eq. L^−1^ during 7 days. Exposure to 372 ± 69 μg STX eq. L^−1^ caused morphological abnormalities in the larvae with severe edema of the eyes, pericardia cavity and yolk sac. Swim bladder failed to inflate. After 2 days of exposure in the 227 and 426 μg STX eq. L^−1^ treatments, a reduced touch response was found in 75% of the larvae. By day 4, 100% of the larvae paralyzed, although the lowest concentration group recovered at day 7. The highest concentration group remained paralyzed. The exposure interval to induce complete paralysis depends on age at which the fish started to be exposed. *D. rerio* larvae exposed at 2 dpf paralyzed after 46 h, at 4 dpf after 8 h and at 6 dpf after 5 h. Older larvae were more sensitive. Those exposed at 2 dpf recovered completely paralysis after 7 days, and the other had recovery rates between 25–50%. This work confirms that toxin uptake occurs in larval fish exposed to STX. Differences in the timing of STX induced paralysis provide preliminary evidence that discrete cell populations in the developing nervous system of larval fish may be differentially susceptible to STX. The rapid effects of STX observed in older larvae may be a result of increased toxin uptake due to gill development and ventilation at these latter development stages. The impact of STX in sensorimotor function and larval morphology were transient and in part reversible, indicating an activity-dependant effect of STX.

Latter, Lefebvre *et al*. [49] studied the effects of an acute exposure (24 h) of STX (50–1,600 μg STX eq. L^−1^) on larvae of Pacific herring (*Clupea harengus pallasi*). Mortality was not significantly higher in exposed larvae compared to control. Nevertheless, 1h EC_50_ varied from 700 μg STX eq. L^−1^ (eggs) to 1,500 μg STX eq. L^−1^ (older larvae), showing an age dependant effect. A 7 day exposure to 400 μg STX eq. L^−1^ was done and larval length was not affected. Normal behavior of fish was registered after 1 to 2 weeks in clean water free of toxin. The onset of STX-induced paralysis was rapid (<1 h) with exposure to STX having a consistent effect on motor activity. At comparable concentrations, herring larvae are less sensitive to toxins than the freshwater zebra fish. These are evidences that marine larvae may have evolved an adaptive resistance to algal toxins. Herring larvae do not show STX-induce morphological abnormalities unlike zebra fish. Paralysis only occurred for few hours and this may be the reason.

### 3.4. Palitoxin

Not much is known in relation to potential toxic effects of PTX on vertebrate larval development. The use of the FETAX assay to estimate the toxicity of palytoxin (PTX) showed that the results were concentration dependant [50]. The test lasted 5 days and during this period, a significant rate of daily embryo mortality was found for the highest concentration used – 370 nM PTX. At day 3, 50% of the embryos exposed to 370 nM of PTX died (LC_50_–370 nM). Many teratogenic individuals were found at this concentration and similar evidences were found for 37 nM PTX at days 4 and 5. Modifications included folding along the antero-posterior body axis, and swelling of the visceral mass. Tail skeletal musculature appeared less compact and with evident loss of muscle fibers. The nervous system was also very sensitive with loss of nerve cells and empty, pigmented areas. No inflammatory responses were found for any of the concentrations used. At the two lowest concentrations (0.37 and 3.7 nM PXT) no significant malformations were found. There is a lack of data concerning marine fish development, but these organisms may be more resistant to the toxin due to co-evolution. Later, a molecular biology approach was also considered to examine the effect of PTX on genes involved in *Xenopus laevis* development [46]. It was demonstrated that the toxin act by inducing modifications in gene expression levels from early stages of *Xenopus* embryogenesis. PTX induced an over-expression in all tested genes (*siamois*, *engrailed*-2, *bmp4* and *myf5*), which are specifically involved in neural and muscular specification and patterning. These genes are directly or indirectly regulated by the Wnt signaling pathway. Wnt proteins form a family of highly conserved secreted signaling molecules that have a crucial role in the regulation of developmental and morphogenetical processes in animals [51]. Mutations or altered expression of genes from this signaling pathway lead to severe consequences in embryos development, consequently affecting the expression of down-regulated genes like those tested in the study [46]. Thus, the altered expression patterns observed apparently corroborate the findings from the FETAX assay earlier performed [46,50].

### 3.5. Brevetoxin

A study on the effects of brevetoxin (BTX) in the development, cardiovascular function and survival of medaka (*O. latipes*) embryos showed that this species is very sensitive to BTX, with the toxin being responsible for extensive fish kills [18]. Fertilized eggs of the model fish were microinjected with PbTX-3 at different doses from 1 to 8.5 ng PbTX-3 egg^−1^. Control eggs hatched at day 10 and all were studied until day 13. The main effects observed were tachycardia (increased heart rate), sustained convulsions and head-first hatching. Higher concentration of PbTX-3 caused an increase in tachycardia at days 4 and 6. The LD_50_ was 4 ng PbTX-3 egg^−1^ at the end of the 13 day exposure time. PbTX-3 affects negatively the development of embryos because it disrupts the differentiation process. Many of the effects observed in the embryos are not seen in intoxicated adults by BTX poisoning.

### 3.6. Ciguatoxin

Fish are the ultimate CTX accumulators. Toxin uptake seems to occur due to phytophagous fish that feed on *G. toxicus*, which transfer the toxin to higher trophic levels up to large predators. Although high levels of CTX lethal to humans may be found in adult fish, there seems to occur a resistance to the toxin by its vectors. Few studies on CTX effects were done on the freshwater *O. latipes*. For instance, Edmunds *et al*. [42] studied the effects of CTX microinjected embryos of this model fish. Embryos were injected with doses ranging from 0.1 to 20 pg CTX egg^−1^. All doses produced cardiovascular, muscular and skeletal deformities and those injected with doses of 1–9 pg CTX egg^−1^ had reduced hatching success (45%) compared to controls (74%). The eggs injected with 0.1 to 0.9 pg CTX egg^−1^ produced 22% of larvae with spinal curvature while this was found in 93% of those exposed to 1–9 pg CTX egg^−1^. All fish with spinal curvature had impaired swimming ability and died within 8 days. The highest doses 10–20 pg CTX egg^−1^ completely inhibited hatching. The lowest observable adverse effect dose was in the range of 0.1–0.9 pg CTX egg^−1^ and similar values were found in the flesh of adult reef fish [52].

A study on the effects of CTX in finfish embryos used fertilized eggs of *O. latipes* microinjected with CTX six to eight hpf with dose ranging from 2.5 to 25 pg CTX egg^−1^ [53]. Control fish initiated heartbeat at 3 dpf and hatching occurred at 9 dpf. By day 4 after injection, embryos injected with 10 pg CTX egg^−1^ showed heart rates significantly lower than those of the control embryos. The highest dose (25 pg) did not show significant differences compared to the control. In terms of behavioral changes, embryos injected with concentrations higher that 10 pg twitched more that the control embryos being the differences statistically significant. The severity of spinal curvature increased due to rising doses of CTX, 16% of eggs exposed to 2.5 pg hatched with deformities and also 80% of those exposed to 25 pg [53]. Spinal curvature induced forced circular swimming, restraining individuals from feeding and eventually resulting in death. Embryos are at a higher risk than adults due to reduced ratio of body mass to toxin load. CTX stored in the adult is not bioavailable but upon transfer to the oocyte becomes available to the embryo over the period of yolk sac absorption and lipid droplet metabolism. Extracts of a toxic Caribbean Great Barracuda (*Sphyraena barracuda*) containing mature eggs provided evidence of CTX transfer from adults to the eggs. Levels in the adults liver were lower (0.12 ng g^−1^ tissue) than in the eggs (0.18 ng g^−1^ tissue). The administration of CTX *via* microinjection does not mimic the natural exposure to the toxin, so it is difficult to extrapolate the observed effects to the natural environment.

Overall, and taking into account the difficulties imposed by the different variables/factors and routes of exposure we may conclude that the toxins analyzed have may have a different impact on fish egg hatching and embryo development. The most toxic seems to be CTX, with effects at the pg level when microinjected in fertilized fish eggs. BTX and DA follow in degree of toxicity. Toxic effects were observed when eggs or embryos were exposed to μg to mg L^−1^ levels of OA or STX, but these represent extreme situations that may rarely be found in Nature. It will be important to perform experiment on the ng to μg levels of the other toxins so as to better estimate the real impact of blooms on the survival and development of fish species.

## 4. Conclusions

Marine toxins have an impact on early life stages of invertebrate and vertebrate species, contributing to significant population changes depending on the severity of the bloom in terms of density and duration. Changes in physiology (respiration, reproduction, growth) and in behavior (vertical migration, feeding activity) may occur depending if the toxin is provided alone or in mixed phytoplankton diets. In some cases, extracts are more toxic than pure toxins showing that phytoplankton produce an array of secondary metabolites with toxicological activity that may have different targets (invertebrates/vertebrates). Toxic effects were observed when eggs, larvae or embryos were exposed to μg to mg L^−1^ levels of toxins but these represent extreme situations that may rarely be found in nature. It will be important to perform experiment on the ng to μg levels of the toxins so as to better estimate the real impact of blooms on the survival and development of invertebrate and vertebrate species. The marine environment is quite dynamic compared to freshwater ecosystems so concentrations of the blooms in some areas of the ecosystem are not common due to the physical dynamics of the media. This might be a reason why the impact of marine toxins produced by phytoplankton is not as deleterious as we find in freshwater systems. Nevertheless, not much is known about the impact on invertebrate and vertebrate reproduction and development of the exposure to many marine toxins *via* natural routes and with ecological relevant concentrations. Also, the development of a resistance to the toxins *via* enhanced detoxification systems by many marine species need to be studied, comparing species from ecosystems not exposed to phytoplankton blooms.

## Figures and Tables

**Table 1:** Marine toxins reviewed in this study, examples of producing organisms, chemical structure and target in the intoxicated organism.

Toxin	Example of producing organism	Chemical type	Target/action

Tetrodotoxin	**Bacteria** *Vibrio* sp. *Serratia marcescens*	Alkaloid	Na^+^ channels/blockage
Nodularin	**Cyanobacteria** *Nodularia spumigena*	Cyclic peptide	Protein Phosphatases/inhibition
Domoic acid	**Diatoms** *Pseudo-nitzschia* spp.	Amino acid	Glutamate receptor/agonist
Okadaic acid	**Dinoflagellates** *Dynophysis* spp.	Polyether	Protein Phosphatases/inhibition
Saxitoxins	*Alexandrium* spp.	Alkaloid	Na^+^ channels/blockage
Palytoxin	*Ostreopsis ovata*	Aliphatic backbone with polyethers	Na^+^/K^+^ pumps/pore forming
Brevetoxins	*Karenia brevis*	Polyethers	Na^+^ channels/
Ciguatoxin	*Gambierdiscus toxicus*	Polycyclic ethers	Na^+^ channels/

**Table 2:** Effects of different marine toxin on the survival of larval stages of several invertebrate species (* values are calculated from data reported by authors) (fil–filaments, TL_50_–lethal time for 50% of the tested organisms, n.a.–not available, n.s.l.–not significant lethality).

Toxin	Invertebrate species	Concentrations tested or cell or filaments density	Toxicity	Reference
**NOD**	*Eurytemora affinis*	0.58 μg NOD mg^−1^ 1.85–22.2 × 10^3^ cells mL^−1^	44–72% mortality	[24]
		0.11 μg NOD mg^−1^ 0.5 × 10^3^ fil mL^−1^	TL_50_–2.5 days	[26]
	*E. affinis* *Acartia bifilosa*	9.2–13.0 μg NOD L^−1^	n.s.l.	[25]
	*A. tonsa* *Temora longicornis*	0.35 pg NOD cell^−1^ 300 μg C L^−1^	n.s.l.	[27]
	*Gammarus zaddachi*	12–101 μg NOD L^−1^	TL_50_–1 day (41 μg NOD L^−1^)	[23]
**DA**	*A. clausi*	0.31 pg DA cell^−1^ 1.9 × 10^4^ cell mL^−1^	n.s.l.	[29]
	*Pecten maximus*	50 ng DA mL^−1^	18.3% survivorship not significantly different from control	[30]
**OA**	*Cancer magister, C. oregonensis, C. productus, C. gracilis, Hemigrapsus nudus, H. oregonensis*	10^3^ cells mL^−1^	Cells not ingested n.s.l.	[32]
	*Artemia salina*	n.a. (cell free media)	LT_50_–1.7 h	[33]
**STX**	*A. salina*	2,000 cells mL^−1^	LT_50_–12 h	[35]
	*Homarus americanus*	n.a. *via* contaminated zooplankton	Maximum lethality–43%	[36]
	*C. magister, C. oregonensis, H. oregonensis, Rhinolithodes wosnessenskii*	1,000 cells mL^−1^	n.s.l.	[37]
	*C. oregonensis, C. magister*	5 × 10^2^ cells mL^−1^ (filtrate)	Reduction of oxygen consumption	[38]
**CTX**	*Artemia* spp.	2–1,000 cells *Artemia*^−1^	LD_50_–2.9 to 42.6 cells	[39]

**Table 3:** Effects of different marine toxin on the egg hatching or embryo development of several fish species (* values are calculated from data reported by authors).

Toxin	Fish species	Concentrations tested	Toxicity	Reference
**DA**	*Danio rerio*	0.16–23.8 ng DA egg^−1^ *	EC_50_ 0.56–1.68 ng DA egg^−1^ *	[10]
**STX**		10–500 μg STX L^−1^	21 d LC_50_ 500 μg STX L^−1^	[41]
	*Clupea harengus*	50–1600 μg STX L^−1^	Not significant	[49]
**BTX**	*Oryzias latipes*	1–8.5 ng PbTX-3 egg^−1^	LD_50_ 4 ng PbTX-3 egg^−1^	[18]
**CTX**		0.1–20 pg CTX egg^−1^	LD_50_ 1–9 pg CTX egg^−1^	[42]
**OA**		0.1–1 μg OA mL^−1^	LD_50_ 0.52 μg OA mL^−1^	[43]

## References

[1] Kao CY Structure-activity relations of tetrodotoxin, saxitoxin and analogues Ann N Y Acad Sci 1986 479 52 67 2434008 10.1111/j.1749-6632.1986.tb15561.x

[2] Scheib H McLay I Guex N Clare J Blaney F Dale T Tate S Robertson G Modeling the pore structure of voltage-gated sodium channels in closed, open, and fast-inactivated conformation reveals details of site 1 toxin and local anesthetic binding J Mol Model 2006 12 813 822 16508760 10.1007/s00894-005-0066-y

[3] Lee MJ Jeong D-Y Kim W-S Kim H-D Kim C-H Park W-W Kim K-S Kim H-M Kim D-S A tetrodotoxin-producing *Vibrio* strain LM-1, from the Puffer fish *Fugu vermicularis radiates* Appl Environ Microbiol 2000 66 1698 1701 10742263 10.1128/aem.66.4.1698-1701.2000 PMC92044

[4] Yu C-F Yu PH-F Chan P-L Yan Q Wong P-K Two novel species of tetrodotoxin-produciong bacteria isolated from toxic marine puffer fishes Toxicon 2004 44 641 647 15501290 10.1016/j.toxicon.2004.07.021

[5] Yan Q Yu PHF Li H-Z Detection of tetrodotoxin and bacterial production by *Serratia marcescens* World J Microbiol Biotechnol 2005 21 1255 1258

[6] Maruyama J Noguchi T Narita H Jeon JK Otsuka M Hashimoto K Occurrence of tetrodotoxin in a starfish, *Astropecten scoparius* Agric Biol Chem 1985 49 3069 3070

[7] Noguchi T Uzu A Koyama K Hashimoto K Occurrence of tetrodotoxin as the major toxinin xanthid crab *Atergatis floridus* Bull Jpn Soc Sci Fish 1983 49 1887 1892

[8] Hanifin CT Brodie ED 3rd Brodie ED Jr Tetrodotoxin levels of the rough-skin newt, *Taricha granulosa*, increase in long-term captivity Toxicon 2002 40 1149 1153 12165318 10.1016/s0041-0101(02)00115-0

[9] Honkanen RE Dukelow M Zwiller J Moore RE Khatra BS Boynton AL Cyanobacterial nodularin is a potent inhibitor of type 1 and type 2A protein phosphatases Mol Pharmacol 1991 40 577 583 1656193

[10] Tiedeken JA Ramsdell JS Ramsdell AF Developmental toxicity of domoic acid in zebrafish (*Danio rerio*) Neurotoxicol Teratol 2005 27 711 717 16061356 10.1016/j.ntt.2005.06.013

[11] Bates SS Gaudet J Kaczmarska I Ehrman JM Interaction between bacteria and the domoic-acid-producing diatom *Pseudo-nitzschia multiseries* (Hasle) Hasle; can bacteria produce domoic acid autonomously? Harmful Algae 2004 3 11 20

[12] Tanti JF Grémeaux T van Obberghen E Le Marchand-Brustel Y Effects of okadaic acid, an inhibitor of protein phosphatases-1 and -2A, on glucose transport and metabolism in skeletal muscle J Biol Chem 1991 266 2099 2103 1846612

[13] Boczar BA Beitler MK Liston J Sullivan J Cattolico RA Paralytic shellfish toxins in *Protogonyaulax tamarensis* and *P. catenella* in axenic culture Plant Physiol 1988 88 1285 1290 16666456 10.1104/pp.88.4.1285 PMC1055754

[14] Kan Y Uemura D Hirata Y Ishiguro M Iwashita T Complete NMR signal assignment of palytoxin and N-acetylpalytoxin Tetrahedron Lett 2001 42 3197 3202

[15] Katikou P Botana LM Chemistry of Palytoxins and Ostreocins Phycotoxins, Chemistry and Biochemistry Blackwell Publishing Ames, IA, USA 2007 75 93

[16] Satoh E Ishii T Nishimura M Palytoxin-induced increase in cytosolic-free Ca^2+^ in mouse spleen cells Eur J Pharmacol 2003 465 9 13 12650827 10.1016/s0014-2999(03)01459-6

[17] Valverde I Lago J Vieites JM Cabado AG *In vitro* approaches to evaluate palytoxin-induced toxicity and cell death in intestinal cells J Appl Toxicol 2008 28 294 302 17604342 10.1002/jat.1278

[18] Colman JR Ramsdell JS The type B Brevetoxin (PbTx-3) adversely affects development, cardiovascular function, and survival in Medaka (*Oryzias latipes*) embryos Environ Health Perspect 2003 111 1920 1925 14644667 10.1289/ehp.6386 PMC1241767

[19] Bagnis R Chanteau S Chungue E Hurtel JM Yasumoto T Inoue A Origins of ciguatera fish poisoning: a new dinoflagellate, *Gambierdiscus toxicus* Adachi and Fukuyo, definitively involved as a causal agent Toxicon 1980 18 199 208 7190330 10.1016/0041-0101(80)90074-4

[20] Labrusse H Matile L Toxicological biotest on diptera larvae to detect ciaguatoxins and various other toxic substances Toxicon 1996 34 881 891 8875775 10.1016/0041-0101(96)00045-1

[21] McMahon BR Tanaka K Doyle JE Chu KH A change of heart: cardiovascular development in the shrimp *Metapenaeus ensis* Comp Biochem Physiol, Part A: Mol Integr Physiol 2002 133 577 587 10.1016/s1095-6433(02)00196-4 12443915

[22] Lopes V Antunes A Martins R Welker M Vasconcelos V Morphological, toxicological and molecular characterization of a benthic *Nodularia* strain isolated from a Portuguese estuary Res Microbiol 2009 10.1016/j.resmic.2009.11.001 19944147

[23] Korpinen S Karjalainen M Viitasalo M Effects of cyanobacteria on survival and reproduction of the littoral crustacean *Gammarus zaddachi* (Amphipoda) Hydrobiologia 2006 559 285 295

[24] Koski M Engstrom J Viitasalo M Reproduction and survival of the calanoid copepod *Eurytemora affinis* fed with toxic and non-toxic cyanobacteria Mar Ecol Prog Ser 1999 186 187 197

[25] Koski M Schmidt K Engstrom.Ost J Viitasalo M Jónasdóttir S Repka S Sivonen K Calanoid copepods fed and produce eggs in the presence of toxic cyanobacteria *Nodularia spumigena* Limnol Oceanogr 2002 47 878 885

[26] Ojaveer E Simm M Balode M Purina I Suursaar U Effect of *Microcystis aeruginosa* and *Nodularia spumigena* on survival of *Eurytemora affinis* and the embryonic and larval development of the Baltic herring *Clupea harengus membras* Environ Toxicol 2003 18 236 242 12900942 10.1002/tox.10120

[27] Kozlowsky-Suzuki B Koski M Hallberg E Wallén R Carlsson P Glutathione transferase activity and oocyte development in copepods exposed to toxic phytoplankton Harmful Algae 2009 8 395 406

[28] Beattie KA Ressler J Wiegand C Kraiuse E Codd GA Steinberg CEW Pflugmacher S Comparative effects and metabolism of two microcystins and nodularin in the brine shrimp *Artemia salina* Aquat Toxicol 2003 62 219 226 12560170 10.1016/s0166-445x(02)00091-7

[29] Maneiro I Iglesias P Guisande C Riveiro I Barreiro A Zervoudaki S Granéli E Fate of domoic acid ingested by the copepod *Acartia clausi* Mar Biol 2005 148 123 130

[30] Liu H Kelly MS Campbell DA Dong SL Zhu JX Wang SF Exposure to domoic acid affects larval development of king scallop *Pecten maximus* (Linnaeus, 1758) Aquat Toxicol 2007 81 152 158 17178425 10.1016/j.aquatox.2006.11.012

[31] Liu H Kelly MS Campbell DA Fang J Zhu J Accumulation of domoic acid and its effects on juvenile king scallop *Pecten maximus* (Linnaeus, 1758) Aquaculture 2008 284 224 230

[32] Perez MF Sulkin SD Palatability of autotrophic dinoflagellates to newly hatched larval crabs Mar Biol 2005 46 771 780

[33] Ajuzie CC Palatability and fatality of the dinoflagellate *Prorocentrum lima* to *Artemia salina* J Appl Phycol 2007 19 513 519

[34] Bag⊘ien E Miranda A Reguera B Franco JM Yasumoto T Oshima Y Fukuyo Y Effect of the toxic dinoflagellate *Alexandrium minutum* on the copepod *Euterpina acutifrons* Harmful and Toxic Algal Blooms Intergovernmental Oceanographic Commission of UNESCO Paris, France 1996 385 388

[35] Zhenxing W Yinglin Z Mingyuan Z Zongling W Dan W Effects of toxic *Alexandrium* species on the surbvival and feeding rates of brine shrimp *Artemia salina* Acta Ecol Sin 2006 26 3942 3947

[36] Robineau B Gagné JA Fortier L Cembella AD Potential impact of a toxic dinoflagellate (*Alexandrium excavatum*) bloom on survival of fish and crustacean larvae Mar Biol 1991 108 293 301

[37] Hinz S Sulkin S Strom S Testermann J Discrimination in ingestion of protistan prey by larval crabs Mar Ecol Prog Ser 2001 222 155 162

[38] Sulkin S Hinz S Rodriguez M Effects of the exposure to toxic and non-toxic dinoflagellates on oxygen consumption and locomotion in stage 1 larvae of the crabs *Cancer oregonensis* and *C. magister* Mar Biol 2003 142 205 211

[39] Kelly AM Kohler CC Tindall DR Are crustaceans linked to the ciguatera food chain? Environ Biol Fishes 1992 33 275 286

[40] Bejarano AC Gulland FM Goldstein T Leger J Hunter M Schwacke LH VanDolah FM Rowles TK Demographics and spatio-temporal signature of the biotoxin domoic acid in California sea lion (*Zalophus californianus*) stranding records Mar Mamm Sci 2008 24 899 912

[41] Oberemm A Becker J Codd GA Steinber C Effects of cyanobacteria toxins and aqueous crude extracts of cyanobacteria on the development of fish and amphibians Environ Toxicol 1999 14 77 88

[42] Edmunds JSG McCarthy RA Ramsdell JS Ciguatoxin reduces larval survivability in finfish Toxicon 1999 37 1827 1832 10519658 10.1016/s0041-0101(99)00119-1

[43] Escoffier N Gaudin J Mezhoud K Huet H Chateau-Joubert S Turquet J Crespeau F Edery M Toxicity to medaka fish embryo development of okadaic acid and crude extracts of *Prorocentrum* dinoflagellates Toxicon 2007 49 1182 1192 17382985 10.1016/j.toxicon.2007.02.008

[44] Casarini L Franchini A Malagoli D Ottaviani E Evaluation of the effects of the marine toxin okadaic acid by using FETAX assay Toxicol Lett 2007 169 145 151 17280804 10.1016/j.toxlet.2006.12.011

[45] Terao K Ito E Yanagi T Yasumoto T Histopathological studies on experimental marine toxin poisoning. I. Ultrastructural changes in the small intestine and liver of suckling mice induced by dinophysistoxin-1 and pectenotoxin-1 Toxicon 1986 24 1141 1151 3564062 10.1016/0041-0101(86)90140-6

[46] Franchini A Casarini L Malagoli D Ottaviani E Expression of the genes *siamois, engrailed-2, bmp4* and *myf5* during *Xenopus* development in the presence of the marine toxins okadaic acid and palytoxin Chemosphere 2009 77 308 312 19683326 10.1016/j.chemosphere.2009.07.027

[47] Gosselin S Fortier L Gagné JA Vulnerability of marine fish larvae to the toxic dinoflagellate *Protogonyaulax tamarensis* Mar Ecol Prog Ser 1989 57 1 10

[48] Lefebvre KA Trainer VL Scholz NL Morphological abnormalities and sensorimotor deficits in larval fish exposed to dissolved saxitoxin Aquat Toxicol 2004 66 159 170 15036871 10.1016/j.aquatox.2003.08.006

[49] Lefebvre KA Elder NE Hershberger PK Trainer VL Stehr CM Scholz NL Dissolved saxitoxin causes transient inhibition of sensorimotor function in larval Pacific herring (*Clupea harengus pallasi*) Mar Biol 2005 147 1393 1402

[50] Franchini A Casarini L Ottavianio E Toxicological effects of marine palytoxin evaluated by FETAX assay Chemosphere 2008 73 267 271 18672264 10.1016/j.chemosphere.2008.06.043

[51] Nusse R Wnt signaling in disease and in development Cell Res 2005 15 28 32 15686623 10.1038/sj.cr.7290260

[52] Poli MA Lewis RJ Dickey RW Musser SM Buckner CA Carpenter LG Identification of Caribbean ciguatoxins as the cause of an outbreak of fish poisoning among US soldiers in Haiti Toxicon 1997 35 733 741 9203298 10.1016/s0041-0101(96)00166-3

[53] Colman JR Dechraoui M-YB Dickey RW Ramsdell JS Characterization of the developmental toxicity of Caribbean ciguatoxins in finfish embryos Toxicon 2004 44 59 66 15225563 10.1016/j.toxicon.2004.04.007

